# Clustering of metabolic syndrome components in a Middle Eastern diabetic and non-diabetic population

**DOI:** 10.1186/1758-5996-2-36

**Published:** 2010-06-08

**Authors:** Alireza Esteghamati, Ali Zandieh, Omid Khalilzadeh, Alipasha Meysamie, Haleh Ashraf

**Affiliations:** 1 Endocrinology and Metabolism Research Center (EMRC), Vali-Asr Hospital, School of Medicine, Tehran University of Medical Sciences, Tehran, Iran; 2 Department of Preventive Medicine, School of Medicine, Tehran University of Medical Sciences, Tehran, Iran; 3 Department of Cardiology, Tehran Heart Center, Tehran University of Medical Sciences, Tehran, Iran

## Abstract

**Background:**

Metabolic syndrome (MetS) encompasses a cluster of coronary heart disease and diabetes mellitus risk factors. In this study, we aimed to elucidate the factors underlying the clustering of MetS components in diabetic and non-diabetic individuals.

**Methods:**

Factor analysis was performed on 2978 (1652 non-diabetic and 1326 diabetic) participants. Entering waist circumference, homeostasis model assessment of insulin resistance (HOMA-IR), triglycerides, high-density lipoprotein-cholesterol (HDL-C) and systolic blood pressure (SBP), we performed exploratory factor analysis in diabetic and non-diabetic individuals separately. The analysis was repeated after replacing triglycerides and HDL-C with triglycerides to HDL-C ratio (triglycerides/HDL-C). MetS was defined by either adult treatment panel III (ATPIII), international diabetes federation (IDF) criteria, or by the modified form of IDF using waist circumference cut-off points for Iranian population.

**Results:**

The selection of triglycerides and HDL-C as two distinct variables led to identifying two factors explaining 61.3% and 55.4% of the total variance in non-diabetic and diabetic participants, respectively. In both diabetic and non-diabetic subjects, waist circumference, HOMA-IR and SBP loaded on factor 1. Factor 2 was mainly determined by triglycerides and HDL-C. Factor 1 and 2 were directly and inversely associated with MetS, respectively. When triglycerides and HDL-C were replaced by triglycerides/HDL-C, one factor was extracted, which explained 47.6% and 38.8% of the total variance in non-diabetic and diabetic participants, respectively.

**Conclusion:**

This study confirms that in both diabetic and non-diabetic participants the concept of a single underlying factor representing MetS is plausible.

## Background

Metabolic syndrome (MetS) encompasses a cluster of coronary heart disease and diabetes mellitus risk factors, including abdominal obesity, glucose intolerance, dyslipidemia and elevated blood pressure [1,2]. Subjects with MetS tend to have high insulin levels, reflecting greater insulin resistance [1-3]. Insulin resistance or hyperinsulinemia has been suggested to be the underlying characteristic of MetS, although a central role for insulin resistance is still controversial [3]. The understanding of how MetS components cluster together can help clinicians interpret the MetS pathophysiology and develop effective strategies for identifying and preventing the inherent risk of coronary heart disease and diabetes mellitus. The multitude of clinical and biochemical alterations resembling the MetS, the strong cross-linkage of involved pathways and multiple feedback mechanisms, complicate the identification of the events which lead to the cascade of disorders that characterize the syndrome [4]. Moreover, statistically strong intercorrelations among various features of MetS, also complicate establishing independent associations using standard multivariate statistical models [2,3]. Factor analysis is potentially a way of advancing this body of research by explaining the correlation between the components of the syndrome in terms of a small set of latent factors, providing insights into the underlying process. Exploratory factor analysis is a hypotheses generation method, in which the number of factors is essentially unknown and has to be determined from data. Extracting one factor implies that a single underlying pathophysiologic mechanism contributes to the appearance of the syndrome. If two or more factors were extracted, the existence of a unique syndrome would be called into question [3].

Previous studies have entered different numbers of variables ranged from 4 to 21 in factor analysis and extracted from 1 to 7 factors [2,5-13]. Some investigations have identified three or four factors, implying a possible heterogeneity of the MetS [2,7,9-11]. Since there appears to be ethnic differences in the expression of MetS [1], different findings may arise on the basis of sample collection. For example, hypertension may not be associated with MetS in American Indians and African Americans [14]. Variations in the extracted factors can also be result of discrepancies in numbers and nature of considered components. For example some studies include insulin levels while others do not; the same is true for uric acid, leptin and waist to hip ratio [2,5-13]. Since factor analysis extracts factors due to the interrelatedness of measured variables, using two or more measures for the same trait (e.g. systolic blood pressure [SBP] and diastolic blood pressure [DBP]) leads to find more factors than expected [15]. These variables compared to other variables are highly associated with each other, thus tend to load on a separate factor. This phenomenon can also be observed with fasting and postprandial glucose or with high density lipoprotein cholesterol (HDL-C) and triglycerides (TG) [5,16].

Factor analysis of the MetS has been the focus of little attention in the Middle Eastern ethnicity; furthermore, the clustering of MetS components is yet to be more explored in the diabetic patients. In this study, for the first time we performed factor analysis on the components of MetS in a large sample of Iranian diabetic and non-diabetic participants. MetS can be defined according to Adult Treatment Panel (ATPIII) [1] or International Diabetes Federation (IDF) criteria [17]. We recently proposed that the optimal waist circumference cut-off points for the diagnosis of IDF defined MetS in Iranian adults would be different from those of other populations [18]. In this study, we also explored the influence of the extracted factors on different definitions of MetS.

## Methods

### Participants

A total of 3023 participants (aged 18-75 yrs), who were referred consecutively from September 2005 to December 2008 to one of the four Tehran University-affiliated health service centers located in east, west, south and center of Tehran, were studied. Participants were enrolled from individuals taking health examinations, or those who accompanied patients. Subjects who needed special concern beyond routine examination for their symptoms (i.e. patients with chronic liver disease, renal, thyroid or adrenal problems) along with those under insulin therapy were not included in the study. Diabetic participants were taking metformin, glibenclamide or both simultaneously for controlling their hyperglycemia. Due to missing data, 45 participants were excluded, resulting in a total of 2978 subjects. Oral informed consent was obtained from all individuals before study commencement. The study was conducted in accordance with Helsinki declaration and was performed in line with considerations, recommended by local ethics review committee of Tehran University of Medical Sciences.

### Procedures

Waist circumference was measured at mid-distance between iliac crest and rib cage and was rounded to the nearest 0.1 cm. The participants were instructed to rest for at least 5 min before having their blood pressure checked twice with at least 5 min interval. Venous blood samples were collected following 12 h overnight fast. Fasting plasma glucose was measured by glucose-oxidase method. TG and HDL-C were determined by enzymatic methods (Parsazmun, Karaj, Iran). Insulin was measured by radioimmunoassay, using an antibody with no cross-reaction against pro-insulin and C-peptide (Immunotech, Prague, Czech Republic). The intra and inter-assay coefficients of variation were lower than 4.3 and 3.4, respectively.

### Definitions

The homeostasis model assessment of insulin resistance (HOMA-IR) was calculated as fasting insulin (U/l) × fasting plasma glucose (mg/dl)/405, as described by Matthews et al. [19]. Diabetes mellitus was diagnosed according to the criteria of American Diabetes Association [20]. The average of two obtained SBPs was used in this study. MetS was defined due to either original ATPIII or IDF declarations. ATPIII criteria allow the diagnosis of MetS when three or more of the following conditions are satisfied: presence of the abdominal obesity (waist circumference ≥102 cm and ≥88 cm in men and women, respectively), elevated blood pressure (SBP ≥130 mmHg and/or DBP ≥85 mmHg), low HDL-C (<40 mg/dl and <50 mg/dl in men and women, respectively), TG ≥150 mg/dl and fasting plasma glucose ≥100 mg/dl (or diabetes) [1,21]. According to original IDF criteria a person with MetS must have abdominal obesity (waist circumference ≥94 cm and ≥80 cm in men and women, respectively) plus any two or more of the following conditions: elevated blood pressure (see above) or treatment of previously diagnosed hypertension, low HDL-C (see above) or on HDL-C therapy, TG ≥150 mg/dl or on TG therapy and fasting plasma glucose ≥100 mg/dl (or diabetes) [17]. MetS was also defined by the modified form of IDF criteria in which the waist circumference cut-offs were substituted by optimal values (waist circumference ≥90 cm in both men and women) for use in Iranian population [18].

### Statistical analysis

Data were analyzed using SPSS software (version 16.0; SPSS Inc., Chicago, USA). To extrapolate our results to the population of Tehran we carried out a complex sample survey analysis. The data were directly weighted for age (10-year strata) and sex distribution of diabetic and non-diabetic residents of Tehran, according to the results of an epidemiological study on patterns of diabetes, conducted by the Ministry of Health [22], and the data of the national censuses of Iran in 2006. To compare the principal characteristics of diabetic and non-diabetic participants, we used the method of general linear modeling in complex sample analysis mode using the F statistic. Comparisons for categorical variables were made by design-based χ^2 ^analysis. The relationship between individual variables was examined using bivariate Pearson's correlation coefficients. Since a large number of analyses were carried out, a *P *<0.01 was considered statistically significant. In order to improve the normality of skewed variables (i.e. HOMA-IR, TG and TG to HDL-C ratio) natural log transformations were used in the subsequent analyses as appropriate. Factor analysis was performed in two separate models. In model 1 waist circumference, SBP, HOMA-IR, TG and HDL-C were selected for analyses. Exploratory factor analysis was performed using principal component analysis, a technique for reducing the number of original variables into fewer latent factors. The variables cluster on the basis of linear correlations that exist among them. Factors with an eigenvalue (the amount of variance attributable to the factor) of greater than one were extracted and transformed by varimax rotation method to enhance interpretation. Varimax method maintains the independence of extracted factors and simplifies the interpretation of analysis by maximizing factor loadings to one or minimizing it to zero. Varimax rotation cannot be performed if factor analysis extracts just one factor. Factor scores, which are estimates of individual factors with the mean and standard deviation equal to 0 and 1, respectively, were determined by regression method. The factor loading of a variable on a factor equals the correlation coefficient between that variable and the factor score. In accordance with several previous reports, factor loadings of ≥|0.40| were considered meaningful for interpretation; because this ensures that the variable shares at least 15% of the variance with the factor [2, 6, 8, 9, 11-13, 15]. Factor analysis was performed in diabetic and non-diabetic participants separately. Binary logistic regression analysis was used to assess the association of factor scores with MetS defined by either ATPIII or original IDF or by the modified form of IDF criteria.

Considering that two of the variables entered in analyses are related to lipid profile (i.e. TG and HDL-C), in model 2 we repeated all aforementioned analyses after substituting ratio of TG to HDL-C for both TG and HDL-C.

## Results

### Descriptive statistics

Sex-specific principal characteristics of our study population in diabetic and non-diabetic participants are presented in Table 1. In men the mean ages ± SEM of diabetic and non-diabetic participants were 53.8 ± 0.5 and 41.1 ± 0.7 years and in women were 54.1 ± 0.4 and 40.0 ± 0.4 years, respectively. There were significant differences between all variables mentioned in Table 1, except insulin concentration. After adjusting for age and sex, in diabetic participants the prevalence of MetS defined by ATPIII, original IDF and modified IDF criteria (74%, 76% and 69%, respectively) was higher than non-diabetic participants (33%, 37%.and 32%, respectively, *P *<0.001)

**Table 1:** Principal Characteristics of the Participants (*n *= 2978).

Characteristic	Men		Women	
	Non-diabetic participants (*n *= 397)	Diabetic participants (*n *= 565)	Non-diabetic participants (*n *= 1255)	Diabetic participants (*n *= 761)
Age (years)	41.1 ± 0.7	53.8 ± 0.5*	40.0 ± 0.4	54.1 ± 0.4*
Waist circumference (cm)	93.2 ± 0.6	99.5 ± 0.4*	90.9 ± 0.4	97.9 ± 0.4*
Fasting plasma glucose (mg/dl)	90.1 ± 0.5	174.3 ± 2.5*	91.5 ± 0.3	171.8 ± 2.1*
Insulin (U/l)	8.1 ± 0.3	8.9 ± 0.2	9.2 ± 0.2	9.0 ± 0.2
HOMA-IR (units)^a^	1.8 ± 0.08	3.7 ± 0.11*	2.1 ± 0.04	3.7 ± 0.09*
TG (mg/dl)	162.2 ± 4.7	208.7 ± 6.8*	133.7 ± 2.1	198.8 ± 4.1*
HDL-C (mg/dl)	46.8 ± 0.6	42.1 ± 0.5*	50.9 ± 0.3	48.5 ± 0.4*
SBP (mmHg)	121.1 ± 0.8	127.8 ± 0.7*	115.2 ± 0.5	131.9 ± 0.7*
Abdominal obesity (%)^b^				
ATP III	22.7	36.0*	54.0	80.7*
IDF	48.5	72.0*	78.6	95.8*
Modified IDF	58.8	85.7*	51.2	76.9*
TG ≥ 150 (%)	43.3	59.9*	33.7	65.8*
Low HDL-C (%)^c^	26.8	46.4*	46.0	57.6*
Elevated blood pressure (%)^d^	35.6	56.8*	27.4	65.7*
MetS (%)^e^				
ATP III	23.0	67.6*	31.3	87.7*
IDF	28.1	66.1*	36.0	89.9*
Modified IDF	31.2	78.1*	26.8	72.9*

Variables are expressed as percentage or mean ± SEM

* *P *<0.001 for the comparison of diabetic and non-diabetic participants. ^a ^Fasting insulin (U/l) × fasting plasma glucose (mg/dl)/405. ^b, c, d and e ^See text for definitions.

Abbreviations: HOMA-IR, homeostasis model assessment of insulin resistance; TG, triglycerides; HDL-C, high density lipoprotein cholesterol; SBP, systolic blood pressure; MetS, metabolic syndrome.

### Univariate associations

In non-diabetic participants, all variables entered in analysis were correlated with each other except HDL-C and SBP. However, in diabetic participants, SBP was merely associated with waist circumference (Table 2).

**Table 2:** Correlation coefficients between variables associated with metabolic syndrome in non-diabetic (n = 1652) and diabetic (n= 1326) participants.

Variable	Waist circumference	HOMA-IR^a^	TG	HDL-C	TG/HDL-C
Non-diabetic participants					

HOMA-IR^a, b^	0.44**				
TG^b^	0.35**	0.27**			
HDL-C	-0.13**	-0.14**	-0.28**		
TG/HDL-C^b^	0.34**	0.28**	0.93**	-0.61**	
SBP	0.37**	0.11**	0.27**	0.01	0.22**

Diabetic participants					

HOMA-IR^a, b^	0.36**				
TG^b^	0.18**	0.25**			
HDL-C	-0.08*	-0.11**	-0.31**		
TG/HDL-C^b^	0.18**	0.24**	0.93**	-0.63**	
SBP	0.09*	0.05	0.03	0.05	0.01

* *P *<0.01; **P <0.001. ^a ^Fasting insulin (U/l) × fasting glucose (mg/dl)/405. ^b ^Log transformed.

Abbreviations: HOMA-IR, homeostasis model assessment of insulin resistance; TG, triglycerides; HDL-C, high density lipoprotein cholesterol; TG/HDL-C, triglycerides to HDL-C ratio; SBP, systolic blood pressure.

### Exploratory factor analysis and logistic regression

**Figure 1:**
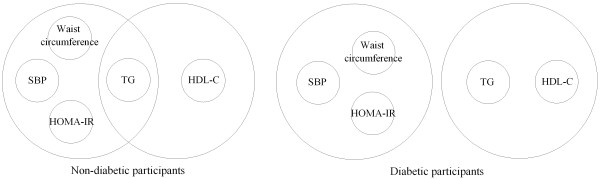
**Factor structure of metabolic syndrome in diabetic and non-diabetic participants due to model 1**. Large circles correspond to factors and small circles to variables. Only the variables with factor loadings ≥|0.40| were selected. Abbreviations: HOMA-IR, homeostasis model assessment of insulin resistance; TG, triglycerides; HDL-C, high density lipoprotein cholesterol; SBP, systolic blood pressure.

Entering waist circumference, HOMA-IR, TG, HDL-C and SBP as variables (components of MetS) in model 1, exploratory factor analysis extracted two factors in both non-diabetic and diabetic participants (Table 3 and Figure 1). Generally, factor 1 was related to waist circumference, HOMA-IR and SBP while factor 2 was mainly determined by TG and HDL-C (Table 3). In non-diabetic participants TG also loaded positively on factor 1. HDL-C loaded positively and TG negatively on factor 2. These two factors together explained 61.3% and 55.4% of the total variance in non-diabetic and diabetic participants, respectively.

**Table 3:** Factor loadings of variables on the selected factors of Model 1 and 2, in diabetic and non-diabetic participants.

Variable	Non-diabetic participants (*n *= 1652)		Diabetic participants (*n *= 1326)	
**Model 1^a^**	**Factor 1**	**Factor 2**	**Factor 1**	**Factor 2**

Waist circumference	0.80*	-0.14	0.76*	-0.12
HOMA-IR^b^	0.56*	-0.35	0.71*	-0.25
TG^b^	0.57*	-0.46*	0.31	-0.69*
HDL-C	0.00	0.89*	0.03	0.81*
SBP	0.75*	0.29	0.50*	0.36
% Total variance explained	36.8	24.5	28.6	26.8

**Model 2**	**Factor 1**		**Factor 1**	

Waist circumference	0.82*		0.74*	
HOMA-IR^b^	0.68*		0.77*	
TG/HDL-C^b^	0.66*		0.59*	
SBP	0.58*		0.21	
% total variance explained	47.6		38.8	

Factors with an eigenvalue ≥ 1 were selected for analysis. * Factor loadings ≥ |0.40|. ^a ^Factor loadings are calculated after varimax rotation of variables in each extracted factor. ^b ^Log transformed.

Abbreviations: HOMA-IR, homeostasis model assessment of insulin resistance; TG, triglycerides; HDL-C, high density lipoprotein cholesterol; TG/HDL-C, triglycerides to HDL-C ratio; SBP, systolic blood pressure.

In model 2, substituting TG and HDL-C with TG to HDL-C ratio led to a single factor structure for both non-diabetic and diabetic participants, which explained 47.6% and 38.8% of variance, respectively (Table 3). HOMA-IR, waist circumference and TG to HDL-C ratio had large factor loadings on the selected factor of model 2 in both diabetic and non-diabetic participants. However, in diabetic participants SBP had lower factor loading than did other variables and thus was less correlated with the factor (Table 3).

Table 4 shows the association of factor scores with MetS, defined by conventional criteria. In model 1, in both non-diabetic and diabetic participants, factor 1 was directly associated with MetS (odds ratio [OR]>1). Nevertheless, factor 2 and MetS were inversely correlated with each other (OR<1). In model 2, the ORs (95% confidence interval) for MetS defined by ATPIII, original IDF and modified IDF were 9.9 (7.8-12.6), 8.4 (6.8-10.4) and 12.4 (9.5-16.0) in non-diabetic subjects and 5.8 (4.6-7.3), 4.4 (3.6-5.4) and 7.4 (5.8-9.4) in diabetic subjects, respectively.

**Table 4:** Odds ratios (95% confidence interval) of factor scores for metabolic syndrome in binary logistic regression in model 1 and 2, Tehran, Iran

Criteria	Non-diabetic (*n *= 1652)		Diabetic (*n *= 1326)	
**Model 1**	**Factor 1**	**Factor 2**	**Factor 1**	**Factor 2**

ATP III	8.9 (7.0-11.2)	0.37 (0.32-0.44)	5.0 (0.4-6.3)	0.38 (0.31-0.46)
IDF	7.4 (6.0-9.1)	0.34 (0.29-0.40)	4.7 (3.8-5.8)	0.66 (0.56-0.78)
modified IDF	10.6 (8.3-13.7)	0.35 (0.29-0.41)	7.0 (5.5-8.9)	0.52 (0.43-0.62)

**Model 2**	**Factor 1**		**Factor 1**	

ATP III	9.9 (7.8-12.6)		5.8 (4.6-7.3)	
IDF	8.4 (6.8-10.4)		4.4 (3.6-5.4)	
Modified IDF	12.4 (9.5-16.0)		7.4 (5.8-9.4)	

*P *< 0.001 for all odds ratios. Odds ratios were determined for every 1 unit increase in factor scores.

Abbreviations: ATP III, adult treatment panel III; IDF, international diabetes federation.

## Discussion

Factor analysis of components revealed two factors in model 1. In diabetic participants the first factor was related to waist circumference, HOMA-IR and SBP while in non-diabetic participants it was also associated with TG. The second factor was determined positively by HDL-C and negatively by TG. Factor 1 and 2 were directly and inversely associated with MetS, respectively. When TG and HDL-C were replaced by a composite measure, which included both of them (i.e. TG to HDL-C ratio), one factor was found, confirming previous observations on the monofactorial structure of MetS [23]. Furthermore, these findings are consistent with the concept that using two or more measures for the same trait results in an overfactored model.

As previously mentioned, variations in sample collection, number and nature of selected components and also in methods used for interpretation of analysis preclude formal direct comparisons, but an overview elucidates some common patterns. In several previous studies insulin resistance loaded on the same factor with measures of obesity including weight, body mass index and waist circumference [2, 5-9, 11, 13]. Furthermore, measures of obesity were more likely to belong to the first extracted factor and to have the highest factor loadings [6-9, 13]. It has been demonstrated that visceral fat via its tendency to release free fatty acids, stimulates hepatic glucose production and accentuates insulin resistance [24]. Moreover, visceral fat cells release some agents to the blood (e.g. plasminogen activator inhibitor-1, interleukin-6 and tumor necrosis factor-α) that are possibly linked to some aspects of insulin resistance [24]. These interrelated pathophysiologic mechanisms, underlying the association of insulin resistance and obesity, may play a principal role in the expression of MetS and its complications including coronary heart disease and diabetes.

MetS is believed to be related to insulin resistance which can be scored by measuring fasting insulin concentration [1-3]. However, due to the fact that in diabetic patients increase in blood glucose and insulin resistance is accompanied by impaired insulin secretion [4], and since there was no significant difference in fasting insulin concentration between diabetic and non-diabetic participants (Table 1); fasting insulin is not a reliable index of insulin resistance in diabetic patients. It has been demonstrated that there is a good correlation between estimates of insulin resistance derived from HOMA-IR and from the euglycemic clamp [25]. Moreover HOMA-IR is a reliable index in diabetic patients and has been widely used as an estimate of insulin resistance in both non-diabetic and diabetic subjects [25,26]. Besides these advantages, HOMA-IR has also some limitations. For instance, in Korean population, the validity of HOMA-IR as a surrogate measure of insulin resistance in lean type 2 diabetic subjects with the insulin secretory defect is disputed [27]. In model 1 of our analyses, HOMA-IR loaded primarily on factor one, but the factor loading of HOMA-IR on factor 2 was -0.35 in non-diabetic participants which is close to the loading threshold, |0.40|. Further, TG was associated with both extracted factors in non-diabetic participants (Figure 1). Many studies have used lower loading thresholds or noted the effect of relaxing the threshold on the findings [6,7,12,15]. Even Cureton and D'Agostino suggested a loading threshold as low as 0.2 for factor interpretation [28]. In model 1, association of HOMA-IR and TG with both extracted factors in non-diabetic participants shows that the two factors are related to each other and are not totally independent. Consistent with our findings, in most studies measures of insulin resistance load on more than one factor, suggesting insulin resistance as a unifying theme underlying the MetS [3]. On the other hand, Shen et al. found that TG and HDL-C are part of the same risk component for MetS and inclusion of both may be redundant [29]. In this regard, TG to HDL-C ratio is reported to be helpful in determining risk of coronary heart disease [30]. In model 2, substitution of TG and HDL-C with TG to HDL-C ratio led to extraction of just one factor. Mannucci et al. reported similar results and revealed that considering TG and HDL-C as two different components of MetS results in overestimation of the role of dyslipidaemia in MetS [5]. These findings suggest that the model 2 of our analyses (one-factor model) is also plausible and support previous studies suggesting one underlying factor (perhaps insulin resistance) for the syndrome [23], the point which is recently substantiated in experimental studies [31].

Insulin resistance and hyperinsulinemia lead to hypertension via a number of mechanisms, including renal sodium and water retention, rennin-angiotensin-aldosterone system, plasma noradrenaline and sympathetic nervous system activity [32,33]. Since subjects' age range is wide (18-75 years) it is more conceivable to use just SBP instead of mean arterial pressure [34]. In model 2, the factor loading of SBP was lowest among components of MetS implying it as the most tenuously linked component. Review of previous studies shows that in most analyses measures of blood pressure load on a unique and separate factor, which does not overlap with other factors of MetS [2,9,11-13], revealing weak relation between blood pressure and other components of MetS [3,29]. Even, such relation may not be evident in some ethnic groups like American Indians and African Americans [14]. Moreover, we found that SBP is less correlated with components of MetS especially in diabetic subjects (Table 2). These findings suggest that blood pressure is less influenced by the common pathophysiologic pathway which underlies the MetS.

Ethnic differences in the expression of MetS are linked to different appropriate waist circumference cut-offs for determination of abdominal obesity. It has been found that Asians in comparison to Caucasians are more prone to obesity related comorbidities even at lower waist circumference values [35]. We recently showed that waist circumference cut-offs recommended by IDF are not appropriate in Iranian population and should change to 90 cm in both men and women [18]. In this study we found that IDF definition modified by our recommended waist circumference cut-offs is strongly associated with extracted factors. As table 4 shows, factor 2 was inversely associated with MetS and therefore had protective roles against it, which seems reasonable because factor 2 was mainly characterized by significant positive factor loading for HDL-C.

The major limitation, relevant to our results interpretation, is use of cross-sectional data. However, consistent with our analysis most previous studies have used cross-sectional study design to evaluate the interrelatedness of MetS components. Cross-sectional analyses provides information at a single point in time, nevertheless progressive and reciprocal mechanisms are likely to be involved in the appearance of MetS. Therefore, longitudinal studies are suggested to improve better our understanding of MetS.

## Conclusions

This study revealed that in both diabetic and non-diabetic individuals the concept of a single underlying factor (pathophysiologic pathway) for the expression of MetS is plausible. Further, we showed that similar to ATPIII and original IDF definitions; modified IDF definition is also strongly associated with identified factors. Nevertheless, among components of MetS blood pressure is less influenced by the common factor and could be also mediated by other pathophysiologic processes.

## Abbreviations

MetS: metabolic syndrome; SBP: systolic blood pressure; DBP: diastolic blood pressure; HDL-C: high density lipoprotein cholesterol; TG: triglycerides; ATPIII: adult treatment panel III; IDF: international diabetes federation; HOMA-IR: homeostasis model assessment of insulin resistance; OR: odds ratio.

## Competing interests

The authors declare that they have no conflict of interests.

## Authors' contributions

AE conceived the study, participated in its design, coordination and acquisition of data. AZ wrote the manuscript and performed the statistical analysis and participated in the acquisition of data. OK helped to draft the manuscript and interpret the data and participated in the acquisition of data. AM helped to perform the statistical analysis. HA helped to draft the manuscript and participated in the acquisition of data. All authors read and approved the final manuscript.
